# The Effect of Benson’s Relaxation Technique on Pain Intensity, Belief, Perception, and Acceptance in adult Hemophilia Patients: A Randomized Controlled Trial

**DOI:** 10.30476/ijcbnm.2021.87937.1471

**Published:** 2021-07

**Authors:** Zahra Molazem, Madineh Alizadeh, Masoume Rambod

**Affiliations:** 1 Community Based Psychiatric Care Research Center, Shiraz University of Medical Sciences, Shiraz, Iran; 2 Department of Nursing, School of Nursing and Midwifery, Shiraz University of Medical Sciences, Shiraz, Iran; 3 Student Research Committee, Shiraz University of Medical Sciences, Shiraz, Iran

**Keywords:** Hemophilia, Pain, Relaxation therapy

## Abstract

**Background:**

Most hemophilia patients experience pain in their lives. Some complementary interventions might affect pain belief. This study aimed to determine the effect of Benson’s relaxation technique on pain intensity, pain belief and perception, and pain acceptance in hemophilia patients.

**Methods:**

In this clinical trial study, 80 hemophilia patients were divided into an intervention (relaxation technique) and a control (routine care) group based on block randomization. This study was conducted in the hemophilia center of Shahid Dastgheib hospital affiliated to Shiraz University of Medical Sciences from October to December 2018. The intervention group listened to a voice containing relaxation technique twice a day for eight weeks. Numeric rating scale, pain belief and perception inventory, and chronic pain acceptance questionnaires were completed at the beginning and eight weeks after the intervention. Data were analyzed by SPSS 21 using independent t-test, Paired-t-test, Chi-square, Mann-Whitney U test, and ANCOVA. P-value <0.05 was considered as significant.

**Results:**

After the intervention, the mean scores of pain intensity, pain belief and perception inventory, and pain acceptance in the intervention group were 4.26±2.17, -13.35±1.50, and 67.24±9.49 and in the control groups were 5.85±2.61, -2±1.70, and 56.57±11.04, respectively. After the intervention, a difference was found between the groups regarding the mean score of pain intensity (P=0.007), pain belief and perception inventory (P<0.001) and its subscales (P<0.05) as well as total pain acceptance (P<0.001).

**Conclusion:**

This study showed relaxation technique can be applied to reduce these patients’ pain intensity, improve their pain belief and perception, and enhance their pain acceptance.

**Trial Registration Number:**

IRCT20180311039037N1.

## INTRODUCTION

Hemophilia is a bleeding disorder caused by congenital deficiency of coagulation factors. ^1 , 2^ Bleeding leads to the joints’ redness, swelling, pain, arthropathy, contracture, and loss of mobility. One of the important consequences of joint hemorrhage in hemophilia patients is pain that more than one third of them suffer from chronic pain. ^3^ Chronic pain exists in all body parts throughout the patients’ lives. Among the adult hemophilia patients referred to the hemophilia center in Shiraz, 71% suffered from pain. ^4^ Pain exists in various body organs, including knees, ankles, shoulders, thighs, elbows, and wrists. Additionally, pain may start and continue at any time even when the patient is asleep. ^5^ Pain not only impacts the patients, but also might affect the patients’ social and family environment and health care systems. ^6^

Pain can result in hopelessness, discomfort, and suffering. ^7^ It is also associated with increased stress, anxiety, and depression in hemophilia patients. ^4^ Pain can also lead to problems in the patients’ social activities, change their social relationships, ^7^ and decrease the health-related quality of life in adult hemophilia patients. ^8^ It was reported that belief of pain affects compatibility with and reaction to it. It was stated that positive pain beliefs and pain acceptance were effective in reduction of pain intensity. ^9^ However, negative beliefs could increase the intensity of chronic musculoskeletal pain. ^10^ Hence, identifying pain beliefs and pain-related behaviors is of utmost importance in management of pain, particularly in patients suffering from chronic pain. ^11^ Pain acceptance is also a key phenomenon in changing the behavior of patients with chronic pain. ^12^ A review of the literature indicated that individuals with high pain acceptance levels reported significantly lower levels of pain, mental disorders, and pain-related disabilities. ^13^

Recently, researchers have paid special attention to Complementary and Integrative Health (CIH), including relaxation, ^14 , 15^ massage therapy, ^16^ reflexology, ^17^ yoga, ^18^ and coping skill training. ^4^ Relaxation techniques are among CIH interventions, which have been considered as psychological interventions. ^19^ Additionally, various nursing measures are being used as complementary treatments to help relieve pain, eliminate the spectrum of damages, and decrease complications. ^20 , 21^ Recently, evidence has indicated that CIH techniques could reduce the intensity of pain among patients. ^22^

Relaxation techniques as examples of nursing-accessible therapies are among CIH interventions, which have received much attention recently. ^19^ Relaxation, as a part of nursing practice, is effective in reduction of mild to moderate pain. It is also an appropriate method for decreasing severe pain and consumption of analgesics. ^23^ The positive outcomes of relaxation include reduced anxiety, supply of energy, reduction of pain resulting from muscular pressure, reduction of pain-related anxiety, and improvement of sleep quality. ^24^

Benson’s relaxation technique was introduced by Herbert Benson (1970), as a simple method for elimination of tension. ^25^ This technique had positive effects on reduction of anxiety and mood disorders, promotion of physical activity, ^21^ and improvement of sleep quality, ^14^ quality of life, pain intensity in hemodialysis patients, ^21^ and cardiac surgery. ^26^ This technique may also be effective in pain intensity, acceptance, and belief in hemophilia patients. However, in recent literature review, no published studies have been conducted on this regard. Nurses are responsible for pain evaluation, using pain measures, and assessment of therapeutic efficacy. The present study aimed to determine the effect of Benson’s relaxation technique on pain intensity, pain belief and perception, and pain acceptance in hemophilia patients.

## METHODS

This randomized controlled clinical trial was conducted in the hemophilia center of Shahid Dastgheib hospital affiliated to Shiraz University of Medical Sciences (SUMS), Shiraz, Iran from October to December 2018. The participants included hemophilia patients who were willing to participate in the study, had the ability to read and write, and had not participated in similar interventional studies including relaxation, massage therapy, and other complementary and alternative integrative medicine during the past three months. The exclusion criteria were not completing the study questionnaires; suffering from known psychological diseases such as depression, anxiety, and psychosis based on their self-report; suffering from other bleeding disorders such as Von Will brand disease, factors I, XII, V, and VII deficiency; not performing the interventional program for three consecutive days; being hospitalized during the study period, and encountering crises such as bereavement or divorce (because these factors could affect the patients’ concentration and the correct performance of Benson’s relaxation technique) based on their self-report. 

Based on a pilot study, α=0.05, power=80% and following formula, and considering the (µ_1_-µ_2_) and δ as 3 and 2.76 for pain intensity, the sample size was estimated 80 subjects. Moreover, by using that study and α=0.05, and power=80%, the (µ_1_-µ_2_) and δ as 2.5 and 3.73 for pain belief, 12.26 and 18.83 for pain acceptance, 70, and 72 subjects were determined for the study. Therefore, the highest sample size (n=80, and 40 patients in each group) was considered for the study.

$n=\frac{\left[{\left(z\frac{\alpha }{2}+\mathrm{z\beta}\right)}^{2}\times \{2{\left(\delta \right)}^{2}\}\right]}{{\left(\mu_{1}-\mu_{2}\right)}^{2}}=\frac{\left[{\left(1.96+0.841\right)}^{2}\times \{2{\left(2.76\right)}^{2}\}\right]}{{\left(3\right)}^{2}}\approx \mathrm{40 ubjects in each group}$

**Figure 1:**
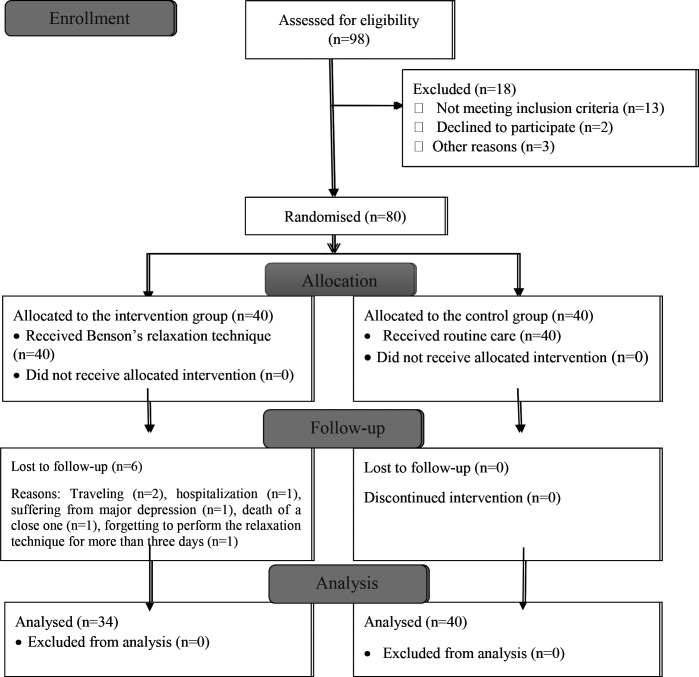
CONSORT flow chart of the participants

At the beginning of the study, 96 subjects were assessed. Eighteen subjects were excluded as a result of not meeting the inclusion criteria (n=13), declined to participate (n=2) and other reasons (n=3). Therefore, 80 subjects were allocated to the intervention and control groups. However, during the study period, six participants were excluded from the intervention group due to traveling (n=2), hospitalization (n=1), suffering from major depression (n=1), death of one of the close ones (n=1), and forgetting the performance of Benson’s relaxation technique for more than three days (n=1) (Figure 1).

After explaining the research objectives and obtaining written informed consents, we divided the patients into two groups using block randomization with block sizes of four. This block was created with a blocked randomization list (https://www.sealedenvelope.com/simple-randomiser/v1/lists). Then, the two groups were required to complete all the questionnaires. After that, Benson’s relaxation program was performed in the intervention group, while the control group received routine care. The two groups completed the questionnaires again at the end of the eighth week. It should be noted that the individuals who collected and analyzed the data were blind to the study groups.

Benson’s relaxation technique was performed by the second author of this study under the supervision of the corresponding author who had previous experience in performance of the technique. The relaxation technique was taught to the patients verbally in a quiet room in the hemophilia ward. An audio file including Benson’s relaxation technique together with instrumental music was played for the patients who were requested to practice the technique as they were listening to the file. In doing so, the patients were required to stay in relaxed position with closed eyes, relax their muscles from the soles of their feet upwards, breath through the nose, pay attention to the sound of their breathing, and say “one” at the time of exhalation. The patients had to practice the technique for 20 minutes and stay in the same position for some minutes after the end of the practice. They were also reminded that they did not have to use alarm rings for checking the end of the 20 minutes. ^14 , 21^ The patients performed the technique in the presence of the researcher and any performance issues were resolved. After the trial, the patients were asked to do the technique for two days. After two days, the patients performed the technique in the presence of the researcher again. Additionally, an educational CD containing the audio file of Benson’s relaxation technique together with instrumental music was given to the patients who were required to practice the technique twice a day (9 A.M. and 5 P.M.), each time lasting for 20 minutes. They were asked to perform this intervention for eight weeks. A self-report form was given to the patients to record their daily performance. In this form, their compliance to Benson relaxation technique (Yes, No) and the times that intervention was done were assessed. The patients had to take the forms to the center at the end of each week for collection by the researcher. Patients were reminded to perform the relaxation technique via telephone contacts through the week. In case the patients forgot to do the technique, they were asked to do so when they remembered. Based on these reported forms, the patients who did not perform the technique for more than three consecutive days were excluded from the study. It should be noted that the researcher gave her phone number to the patients, so that they could ask their possible questions and problems.

The control group received the routine care including factor injection training, control of reaction to pharmacological treatments, control of bleeding and the related pharmacological measures, periodical assessment of laboratory tests, dental care, physiotherapy for the injured joints and muscles, and periodical examinations by physicians. However, they underwent no relaxation interventions. To prevent bias, we provided the control group participants with no information regarding the intervention and the subjects in the intervention group were asked not to provide or give the CD containing Benson’s relaxation technique to the control group or others until they were told. Yet, the control group subjects were given a CD containing Benson’s relaxation technique at the end of the eighth week. 

The study data were collected using a demographic information form information about age, educational level, marital status, occupation, type of hemophilia, history of hemophilia in the family, absence from work due to bleeding and hemophilia problems, severity of hemophilia, having hepatitis, number of bleeding area during the past six months, bleeding areas during the past six months, and pain zones within the past six months; a Numeric Rating Scale (NRS); pain belief and perception inventory; and Chronic Pain Acceptance Questionnaire (CPAQ). 

NRS was used to assess the intensity of pain. NRS is a 10-cm scale ranging from 0 (no pain) to 10 (the worst possible pain). ^27^ Patients mark their pain intensity on this scale. The test-retest reliability of the scale was reported to be 0.96 and 0.95 in literate and illiterate rheumatoid arthritis patients, respectively. ^27^ Besides, the validity of NRS with Visual Analogue Scale was reported to be 0.86-0.95 in patients with rheumatism and other chronic diseases. ^28^ The reliability of Persian version of NRS was assessed by Rambod et al. in a study conducted on hemodialysis patients in Shiraz in 2014 using test-retest method (r=0.94). ^21^

Pain belief and perception inventory was designed by Williams and Thorn (1989). It consisted of 16 items and could be scored via a 4-point Likert scale ranging from -2 (total disagreement) to +2 (total agreement). This inventory consisted of four subscales, namely belief about duration of pain, belief about the stability of one’s pain, belief in pain self-blame, and belief in pain as mysterious and unexplained. Higher scores in this inventory represented the individuals’ deeper belief in pain. Williams and Thorn approved the validity of the inventory by factor analysis. Based on this factor analysis, three factors including “self-blame, perception of pain as mysterious, and beliefs about the duration of pain” were fit to the data, and these factors accounted for 94% of the total variance. Moreover, the reliability of self-blame, perception of pain as mysterious, and beliefs about the duration of pain subscales were approved by Cronbach’s alpha coefficient as 0.65, 0.80, and 0.80, respectively. ^29^ The validity of the Persian version of the pain belief and perception inventory were demonstrated by Asghari Moghadam et al. using factor analysis. This analysis showed four factors. They reported Cronbach’s alpha coefficient of these factors between 0.70 and 77.0. ^30^ In the present study, also, the reliability of the pain belief and perception inventory was confirmed by Cronbach’s alpha=0.76.

CPAQ which was developed by Geiser in 1992 and contains 34 items. ^31^ Then, McCracken, Vowles, and Eccleston reduced the items to 20 in 2004. CPAQ items assess pain acceptance. This questionnaire consists of two subscales, namely activity engagement (11 items) and pain willingness (9 items). The items were scored via a 7-point Likert scale ranging from 0 (never true) to 6 (always true). The scores of the two subscales were summed up and the total score could range from 0 to 120, with higher scores representing higher chronic pain acceptance. The validity of CPAQ was approved by factor analysis, and four factors accounted for 46.8% of the total variance. In addition, the reliability of this questionnaire was approved by Cronbach’s alpha=0.78. ^32^ The reliability and validity of this questionnaire with 20 items were confirmed by Mesgarian et al. in an Iranian study published in 2010. ^33^ Accordingly, its reliability was confirmed with Cronbach’s alpha coefficient of 0.85 and test-retest coefficient of 0.71. The validity of Persian version of this questionnaire was determined using factor analysis. Firstly, the data of 245 subjects were collected; it was shown that these participants were suitable for conducting factorial analysis. Based on this analysis, 40.5% of the variance of CPAQ was determined by these items. Additionally, its convergent validity with pain self-efficacy questionnaire was proved. Its divergent validity was also verified by computing the correlation with physical disability, depression, anxiety, pain intensity, and catastrophe. ^33^ In the present study, the reliability of the CPAQ was approved by Cronbach’s alpha=0.86.

This study was approved by the Ethics Committee of Shiraz University of Medical Sciences (Code: IR.SUMS.REC.1396.15274). The study was explained to patients who were required to sign written informed consent for taking part in the research. They were reassured about the confidentiality of their information and the voluntary nature of the study. Permission to use the Persian version of the study questionnaires was also obtained from its designers (Asghari Moghadam and Mesgarian).

The data were analyzed using SPSS statistical software, version 21. Independent t-test and chi-square test were used to assess differences between the two groups regarding demographic characteristics. Additionally, independent t-test and Mann-Whitney U test were used to compare the study groups’ pre-and post-intervention. Paired t-test also was used. Moreover, ANCOVA was used to control the effect of the confounding variables (total score of pain acceptance) on the post-test. P<0.05 was considered to be statistically significant.

## RESULTS

The mean age of the participants was 29.87±8.01 years; the mean age of the intervention participants was 30±9.16 and 29.73±7.05 years in the control groups. All patients were male. Half of the subjects in the control group were single and half of those in the intervention group were married. Moreover, most participants in the two groups were employed. Indeed, more than half of the participants in the control and intervention groups had diploma and below educational levels, respectively (Table 1).

**Table1:** Demographic and clinical characteristics of the patients in the intervention and control groups

Variables	Groups		P value*
	Control N(%)	Intervention N(%)	
**Education level**			
Diploma and Below	25 (62.50)	18 (53)	0.74
Academic	15 (37.50)	16 (47)	
**Marital status**			
Single	20 (50)	16 (47)	0.61
Married	17 (42.50)	17 (50)	
Divorced	3 (7.50)	1 (3)	
**Occupation**			
Jobless	6 (15)	4 (11.80)	0.69
Employed	34 (85)	30 (88.20)	
**Absence from work due to bleeding and hemophilia problems**			
Yes	28 (70)	25 (73.50)	0.62
No	12 (30)	9 (26.50)	
**Family history of hemophilia**			
Yes	29(72.50)	27 (79.40)	0.49
No	11(27.50)	7 (20.60)	
**Type of hemophilia**			
A	33 (82.50)	28 (82.40)	0.98
B	7 (17.5)	6 (17.60)	
**Severity of hemophilia**			
Moderate	14 (35)	6 (17.60)	0.09
Severe	26 (65)	28 (82.40)	
**Having hepatitis**			
Yes	5 (12.50)	7 (20.60)	0.34
No	35 (87.50)	27 (79.40)	
**Number of bleeding area during the past six months**			
1	17 (42.50)	11 (32.40)	0.37
≥ 2	23 (57.30)	23 (67.60)	
**Bleeding area during the past six months**
Knee joint	8 (20.00)	7 (20.60)	
Elbow joint	1 (2.50)	2 (5.90)	
Ankle joint	2 (5.00)	0 (00)	
Knee & Ankle joints	10 (25.00)	11 (32.40)	0.75
Knee & Pelvic joints	5 (12.50)	6 (17.60)	
Ankle & Elbow joints	2 (5.00)	2 (5.90)	
All joints^a^	3 (7.50)	1 (3)	
Knee joint & Nose	2 (5.00)	2 (5.90)	
Ankle joint & Gastrointestinal system	1 (2.50)	1 (2.90)	
Muscles	1 (2.50)	2 (5.90)	
Nose	2 (5.00)	0 (00)	
Gastrointestinal system	1 (2.50)	0 (00)	
Other	2 (5.00)	0 (00)	
**Pain area during the past six months**			
Knee	9(22.50)	12 (35)	0.72
Elbow	3 (7.50)	2 (6)	
Ankle	2 (5)	1 (3)	
Pelvis	1 (2.50)	1 (3)	
All joints^a^	1 (2.50)	1 (3)	
Muscles	1 (2.50)	2 (6)	
Nose	1 (2.50)	0 (0)	
Gastrointestinal system	1 (2.50)	0 (0)	
More than two areas	18 (45)	15 (44)	
Other	3 (7.50)	0 (0)	

a All joints including joint’ knee, elbow, ankle, pelvis, and femur;

* Chi-square

Most of the patients in the two groups suffered from severe hemophilia and were absent from work due to bleeding during the past six months. Nearly two thirds of the patients in both study groups suffered from hemophilia A and had the family history of the disease. In addition, approximately one-eighth of the patients in the control group and one-fifth of those in the intervention group had hepatitis. The two groups’ demographic and clinical characteristics are presented in Table 1. Accordingly, the two groups were homogeneous with respect to demographic and clinical variables.

The participants of this study had experienced bleeding in their knees, elbows, ankles, femur, and pelvis in the past six months. They had also experienced gastrointestinal, muscular, and mucosal bleeding. Almost half of the participants in both study groups had pain in more than two areas, with nearly one third of the patients in the two groups reporting pain in their knees.

**Figure 2:**
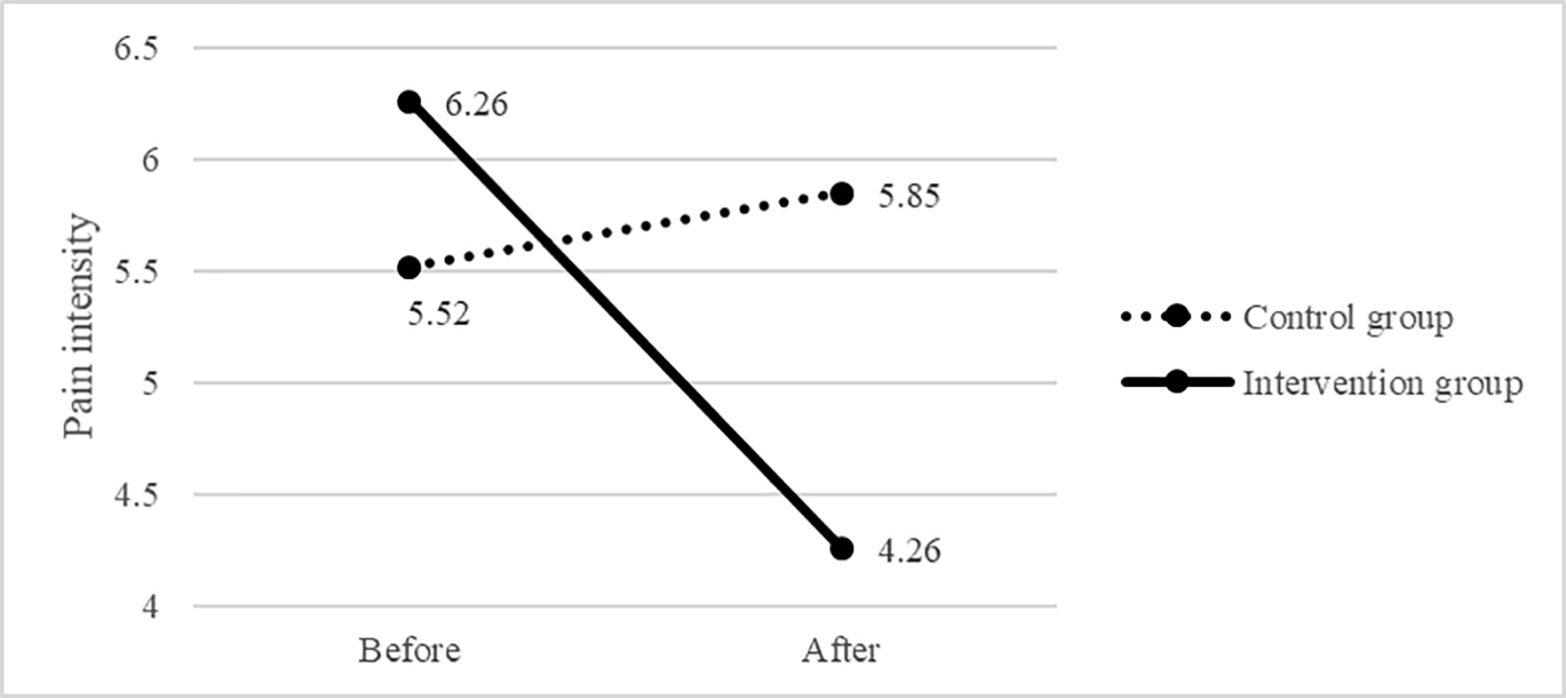
Trend of changes in the mean score of pain intensity in the two groups before and after the intervention

Before the intervention, the mean intensity of pain was 5.82±2.63 in the control group and 6.26±2.56 in the intervention group. No significant difference was observed between the two groups with regard to pain intensity before the intervention (t=-0.72, P=0.47). However, after the intervention, the mean intensity of pain was 5.85±2.61 in the control group and 4.26±2.17 in the intervention group. A significant difference was found between the two groups regarding the mean score of pain intensity after the intervention (t=2.80, P=0.007) (Figure 2).

**Figure 3:**
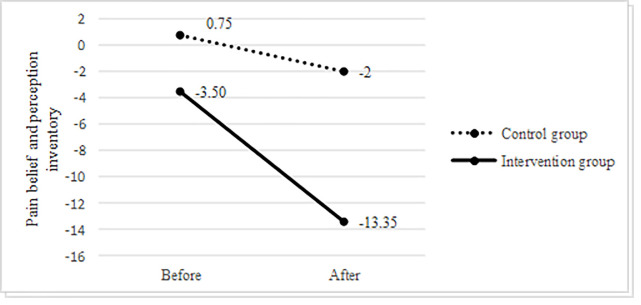
Trend of changes in the two groups’ scores of pain belief and perception inventory before and two months after the intervention

There was no significant difference between the two groups regarding pain belief and perception inventory and its subscales before the intervention (P>0.05) (Table 2). However, a significant difference was observed between the two groups with regard to pain belief and perception inventory and its subscales after the intervention (P<0.05). The trend of changes in the two groups’ scores of the pain belief and perception inventory before and after the intervention is presented in Figure 3.

**Table2:** The mean scores of pain belief, pain acceptance, and their subscales in the two groups before and after the intervention

Pain belief and acceptance		Time	Before the intervention	After the intervention	P value
		Group	Mean±SD	Mean±SD	
Total pain belief		Control	0.75±1.70	-2±1.70	0.004****
		Intervention	-3.50±1.70	-13.35±1.50	<0.001****
		P value	0.8*	<0.001**	
Pain belief subscales
	Belief about the duration of pain	Control	0.45±0.59	-0.07±0.61	0.09****
		Intervention	-0.47±0.65	-2.53±2.83	<0.001****
		P value	0.29*	0.005**	
	Belief in pain self-blame	Control	0.05±0.57	-1.35±0.55	<0.001****
		Intervention	-1.18±0.49	-2.9±0.44	0.001****
		P value	0.13**	0.03**	
	Belief about the stability of one’s pain	Control	-0.15±0.66	0.20±0.68	0.4****
		Intervention	-0.79±0.77	-2.56±0.69	0.005****
		P value	0.52*	0.006*	
	Belief in pain as mysterious	Control	0.40±0.57	-0.80±0.58	0.004****
		Intervention	-1.06±0.72	-5.30±0.33	<0.001****
		P value	0.11*	<0.001**	
Total pain acceptance		Control	56.17±9.97	56.57±11.04	0.56****
		Intervention	62.50±13.60	67.24±9.49	0.004****
		P value	0.02*	<0.001***	
Pain acceptance subscales
	Activity engagement	Control	38.80±12.70	41.10±12.30	0.005****
		Intervention	42.47±10.50	48.17±9.60	<0.001****
		P value	0.19*	0.008*	
	Pain willingness	Control	17.05±11.13	15.47±10.70	0.02****
		Intervention	20.03±9.32	19.06±7.41	0.44****
		P value	0.26*	0.10*	

* Independent t-test,

** Mann-Whitney U test,

*** ANCOVA,

**** Paired t-test

**Figure 4:**
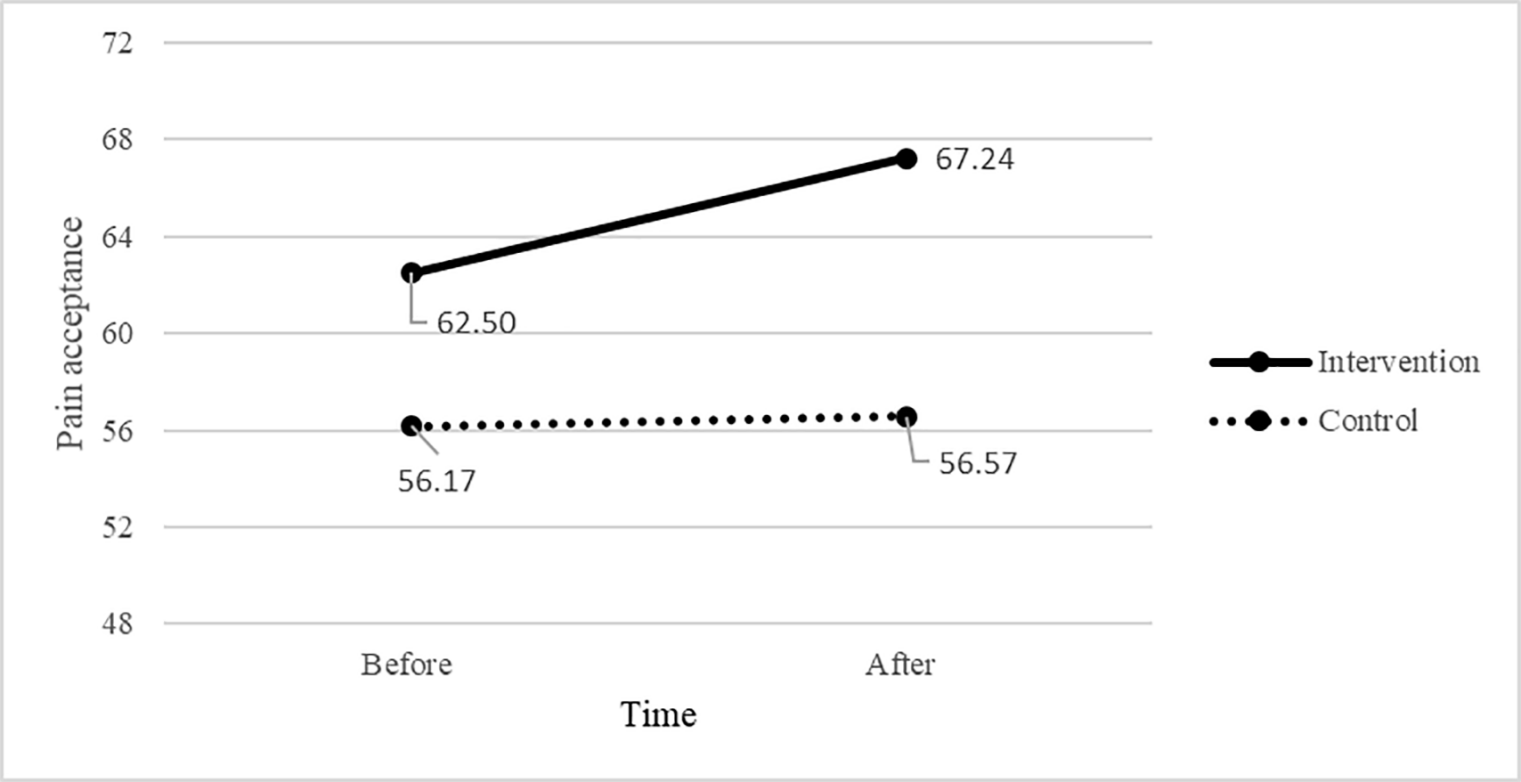
Trend of changes in the two groups’ pain acceptance scores before and two months after the intervention

There was a significant difference between the two groups regarding the mean score of pain acceptance before the intervention (P=0.02) (Table 2). Therefore, this confounding variable was controlled by ANCOVA. Nonetheless, no significant difference was observed between the two groups regarding the subscales of pain acceptance, i.e. activity engagement and pain willingness, before the intervention (P>0.05). However, a significant difference was found in the scores of total pain acceptance and one of its subscales named activity engagement in the two study groups after the intervention (P<0.05), while this was not the case concerning the mean score of pain willingness (P>0.05). The mean scores of pain acceptance in the two groups before and after the intervention are presented in Figure 4.

## DISCUSSION

The study showed that Benson’s relaxation technique decreased the pain intensity and improved the pain belief, perception, and acceptance in hemophilia patients. In this study, the hemophilia patients who performed Benson’s relaxation technique reported lower pain intensity compared to the control group. Similarly, it was shown that Benson’s relaxation technique reduced pain intensity in acute or chronic conditions. ^22 , 26^ It seems that Benson’s relaxation technique helps hemophilia patients accept their illness as well as their unchangeable life conditions. It reduces negative thoughts and emotions associated with pain and, consequently, decreases pain intensity. ^34^ Similar to meditation, relaxation increases attention on the present moment, declines tension by preventing the transfer of pain to the spinal cord, and relaxes the muscles. Indeed, stimulation of endorphin secretion in the brain as the result of performing relaxation leads to muscle relaxation followed by decreased pain intensity among patients. ^21^

In the current study, Benson’s relaxation technique improved the scores of the pain belief and perception inventory and its subscales in hemophilia patients. It has been reported that psycho-education/physiotherapy interventions could affect the pain belief and attitude. ^35^ Based on the researcher experience (second author) and considering the chronic and continuous nature of pain in hemophilia patients, they imagine that there is no definite treatment for their disease and that they have to stand the pain all through their lives. Relaxation therapy probably helps hemophilia patients put aside their previous beliefs about pain and form new positive beliefs. These positive beliefs promote the individuals’ compatibility and daily functions ^30^ and play a critical role in pain coping strategies. ^36^ Strategies for coping with pain, such as massage therapy and relaxation techniques can decrease the intensity of pain and disability and increase compatibility by reducing fear and catastrophizing thoughts about pain. ^36^

The present study revealed a significant difference between the two groups with respect to the mean scores of chronic pain acceptance and its subscales after the intervention. It has also been reported that acceptance- and mindfulness-based interventions might help the patients cope with pain. ^37^ It was indicated that modified mindfulness-based cognitive therapy was effective in acceptance of pain in patients with fibromyalgia. ^34^ Overall, pain acceptance interventions were effective in reduction of pain experience. ^38^

One of the strengths of this study was that performance of Benson’s relaxation technique improved the pain intensity, belief, perception, and acceptance. Thus, this technique can be recommended as a complementary method of pain intensity reduction and increase in pain belief and acceptance in hemophilia patients. 

One of the limitations of this study was that the participants were selected from a single center, so the results cannot be generalized to other regions of the country. Another study limitation included short intervention and follow-up periods. Further longitudinal studies are suggested to be conducted on the issue. 

In assessment eligibility stage of the trial, 13 participants were declined as a result of the study inclusion criteria. As this decline was inevitable, it is suggested that this intervention should be used for all of the hemophilia patients.

## CONCLUSION

The study results indicated that Benson’s relaxation technique reduced the pain intensity and improved the pain belief, perception, and acceptance in hemophilia patients. Further studies are suggested to be conducted on the issue to improve evidence-based practice. This study failed to assess the predictors of pain intensity, belief, perception, and acceptance in hemophilia patients; therefore, another study in this regard is recommended. Moreover, the effect of Benson’s relaxation technique on hemophilia patients’ outcomes such as stress and anxiety levels, quality of life, physical activities, and quality of sleep is suggested to be assessed in another study. In addition, the effect of Benson’s relaxation technique on illness perception and usage of painkillers or opium is recommended to be investigated in future study. 
